# *Francisella tularensis* PCR detection in Cape hares (*Lepus capensis*) and wild rabbits (*Oryctolagus cuniculus*) in Algeria

**DOI:** 10.1038/s41598-022-25188-0

**Published:** 2022-12-12

**Authors:** Imene Ammam, Camille D. Brunet, Nouria Boukenaoui-Ferrouk, Julien Peyroux, Sylvie Berthier, Jean Boutonnat, Karim Rahal, Idir Bitam, Max Maurin

**Affiliations:** 1 Institute of Veterinary Sciences, University of Blida 1, Blida, Algeria; 2 Laboratory of Biodiversity and Environment: Interactions, Genome, University of Sciences and Technology Houari Boumedienne, Algiers, Algeria; 3 University Grenoble Alpes, CNRS, TIMC, 38000 Grenoble, France; 4 Laboratory of Research on Arid Zones Lands (LRZA), Faculty of Biological Sciences (FSB), Houari Boumediene University of Science and Technology (USTHB), BP 32, 16111 Bab Ezzouar, Algiers Algeria; 5 Grenoble Alpes University Hospital, 38000 Grenoble, France; 6 Superior School of Food Sciences and Food Industries of Algiers, El Harrach, Algeria

**Keywords:** Ecology, Microbiology, Zoology, Diseases

## Abstract

Tularemia is a zoonosis caused by the bacterium *Francisella tularensis*. Leporids are primary sources of human infections in the northern hemisphere. Africa is classically considered free of tularemia, but recent data indicate that this dogma might be wrong. We assessed the presence of this disease in wild leporids in Algeria. Between 2014 and 2018, we collected 74 leporids carcasses from spontaneously dead or hunted animals. *Francisella tularensis* DNA was detected by specific real-time PCR tests in 7/36 (19.44%) Cape hares (*Lepus capensis*) and 5/38 (13.15%) wild rabbits (*Oryctolagus cuniculus*). Known tularemia arthropod vectors infested half of the PCR-positive animals. At necropsy, *F. tularensis*-infected animals presented with an enlarged spleen (n = 12), enlarged adrenal glands (12), liver discoloration (12), hemorrhages (11), and pneumonia (11). Immunohistological examination of liver tissue from one animal was compatible with the presence of *F. tularensis*. Our study demonstrates the existence of tularemia in lagomorphs in Algeria. It should encourage investigations to detect this disease among the human population of this country.

## Introduction

Since antiquity, wildlife has been an essential source of infectious diseases transmissible to humans^1,2^. These infections, referred to as zoonoses, constitute a significant public health problem on all continents^2^. The importance of such diseases in humans and animals is increasingly recognized, and more attention in this area is needed^2^. Most emerging infectious diseases in humans are zoonoses^3^. The COVID-19 pandemic is the most recent example^4,5^. Wildlife represents a significant and often unknown reservoir for emerging pathogens and a source for the re-emergence of previously controlled zoonoses (e.g., brucellosis)^6,7^. In addition, such infectious agents also have harmful effects on domestic animals and wildlife, with substantial economic consequences and a threat to species preservation^7,8^. Zoonotic pathogens that infect both domestic and wildlife animals are more likely to emerge as human pathogens^7^.

An excellent example of an emerging zoonotic agent with many different modes of transmission to humans is *Francisella tularensis*, the causative agent of tularemia^2,9^. This Gram-negative, facultative intracellular bacterium is highly infectious and considered a biological threat agent^10^. It is mainly distributed in Northern hemisphere^11^. Two distinct subspecies that cause tularemia are recognized. *Francisella tularensis* subsp. *tularensis* (type A strains), the most virulent subspecies, is almost exclusively found in North America^12^. A few type A strains have been isolated from environmental sources and arthropods in Slovakia and Austria^13^. *Francisella tularensis* subsp. *holarctica* (type B strains) is endemic throughout the Northern hemisphere^14^. Recently, this subspecies has been isolated from tularemia patients and ringtail possums in Australia, especially in Tasmania and Sydney^15,16^.

Many wild animal species can be infected or are carriers of *F. tularensis*, but their epidemiological role remains unclear^11^. *Francisella tularensis* has been isolated from more than 250 species, including lagomorphs, rodents, insectivores, carnivores, ungulates, marsupials, birds, amphibians, fishes, and invertebrates^11,17^. However, lagomorphs and small rodents are considered as the primary sources of human infections^18^. Moreover, lagomorphs (especially hares) often die from *F. tularensis* infection and thus are deemed suitable sentinels for tularemia surveillance^17^. In the Northern hemisphere, human tularemia cases frequently occur through contact with infected animals^19,20^. However, other common modes of infection include arthropod bites (mainly ticks), inhalation of contaminated aerosols, ingestion of contaminated water or food, and contact with contaminated water or soil^21^.

Africa has been considered for decades as free of tularemia^21,22^. A suspected *F. tularensis* bacteremia was reported from Sudan, but the isolated bacterial strain was not fully characterized^23^. Besides, positive *F. tularensis* serological tests have been detected in febrile patients from Kenya^24^. However, there was no confirmation of *F. tularensis* infection by the isolation of bacteria or molecular tests. Finally, *Francisella sp.* DNA was detected in ticks collected on camels in Egypt, but *F. tularensis* was not identified among *Francisella sp*. positive ticks^20^. Altogether, we can conclude that, while strongly suspected, there is currently no definite confirmation of the presence of *F. tularensis* in Africa.

In Northern Algeria, from December 2012 to March 2013, much higher mortality rates than usual were observed in hares and wild rabbits, suggesting an epizootic. Although Algeria is currently not considered endemic for tularemia, this study was conducted to investigate the presence of this disease in wild leporids. Our investigations were performed in Northern Algeria for 4 years following the epizootic, in areas where an excess of mortality in lagomorphs was observed.

## Results

### Study area

**Figure 1:**
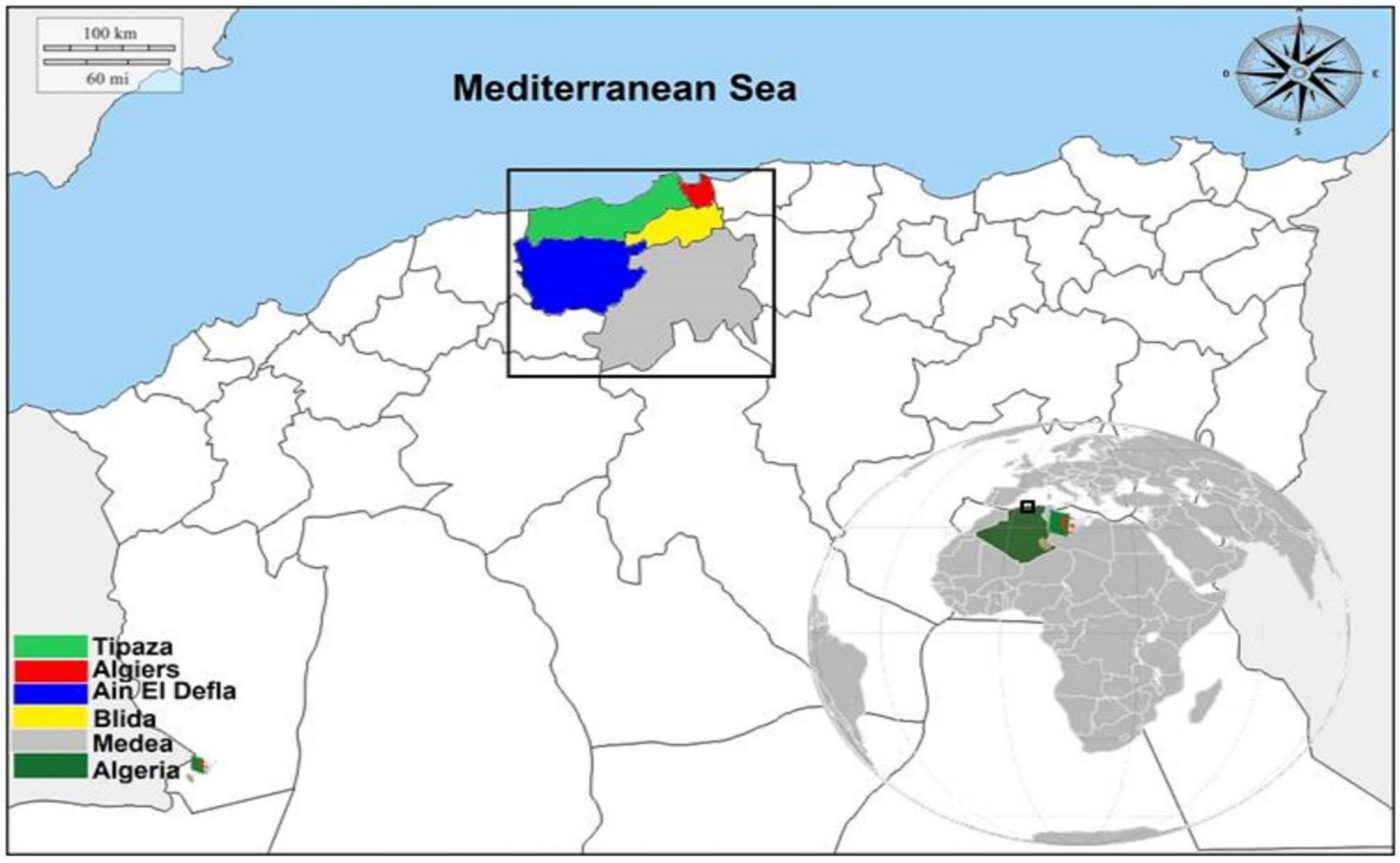
Map of five localities of wild leporids collected in the study area. (The map was generated in paint standard software from a virgin map figure. The map of Algeria is freely accessible at this internet link https://d-maps.com/m/africa/algeria/algerienord_fr/algerienord_fr19.gif.

This study was conducted in the central part of Northern Algeria, which is part of the Tell Atlas mountain range and located on the shores of the Mediterranean Sea. It covers five wilayas (provinces): Tipaza (36° 35′ 31″ N, 2° 26′ 58″ E), Ain El Defla (36° 15′ 55″ N, 1° 58′ 13″ E), Medea (36° 16′ 03″ N, 2° 45′ 00″ E), Blida (36° 29′ 00″ N, 2° 50′ 00″ E), and Algiers (36° 46′ 34″ N, 3° 03′ 36″ E) (Fig. 1). These provinces include mountains, hills, and plains. The climate is of Mediterranean type with an average rainfall of 600–800 mm per year and an average annual temperature of 18 °C near the coast and 25 °C in the inner wilayas. A diverse fauna, including sedentary terrestrial birds (e.g., rapacious, sparrow, partridge), migratory birds, reptiles, and mammals (e.g., wild boars, carnivores, leporids, and rodents), characterizes this region.

### Animal specimens and real-time PCR (qPCR) testing

During the period 2014–2016 and in 2018, we collected 74 wild leporids (36 Cape hares and 38 wild rabbits), which were shot by hunters (n = 60) or found dead (n = 14). The characteristics of the animals (age, sex, location and period of collection) are summarized in Table 1. In total, 74 spleen and 20 liver specimens were tested for the presence of *Francisella* DNA using three qPCRs assays.

**Table 1:** Characteristics of collected wild rabbits and Cape hares.

Collection region	Collection period	Leporids number collected		Sex number collected		Age number collected	
		Hares	Wild rabbits	Female	Male	Mature	Juvenile^a^
Tipaza	Aug 2014–Jan 2016	27	32	18	41	41	18
Blida	Mar–Apr 2018	4	4	2	6	7	1
Ain El Defla	Oct–Dec 2014	2	2	1	3	4	0
Medea	Jul–Nov 2014	2	0	0	2	2	0
Algiers	Mar 2018	1	0	1	00	1	0

^a^ The positive juvenile was a wild rabbit.

The ISFtu2-qPCR targets the multi-copy IS*Ftu*2 insertion sequence. It is highly sensitive but lacks specificity because this *ISFtu2* is found in several *Francisella* species. The Tul4-qPCR targets the gene encoding the membrane protein Tul4, which is more specific to *F. tularensis* but also detects *F. novicida*^25^. The Type B-qPCR targets a particular DNA fragment specific to *F. tularensis* subsp. *holarctica*, the only subspecies causing tularemia in Eurasia^26^.

Overall, 60 samples were negative for the three qPCR tests, 22 were positive only for ISFtu2-qPCR (level 1), ten were positive for both ISFtu2-qPCR and Tul4-qPCR (level 2), and two were positive for all three qPCR tests (level 3). Among the 74 spleen samples, 48 were negative, 15 graded level 1, nine level 2, and two level 3. For the 20 liver samples, 12 were negative, seven level 1, one level 2, and none level 3 (Table 2).

**Table 2:** qPCR test results for hares and wild rabbits and their organ samples.

Positive qPCR tests	Hares			Wild rabbits			Both animals	
	Positive/tested			Positive/tested			Positive/tested	
	Animals (n = 36)	Spleen (n = 36)	Liver (n = 13)	Animals (n = 38)	Spleen (n = 38)	Liver (n = 07)	Animals (n = 74)	Organs (n = 94)
Level 3	1	1/36	0/13	1	1/38	0/7	2	2/94
Level 2	6	5/36	1/13	4	4/38	0/7	10	10/94
Level 1	8	6/36	5/13	11	9/38	2/7	19	22/94
None	21	24/36	7/13	22	24/38	5/7	43	60/94

Level 3: ISftu2, Tul4, and Type B qPCR positive; Level 2: ISftu2 and Tul4, but not Type B qPCR positive; Level 1: only ISftu2 qPCR positive; none: no qPCR positive.

### Tularemia infected animals

Since no *F. tularensis* strain could be isolated from animal samples, the animals were classified (see “Materials and methods” section) as “probable” tularemia case (at least one level 3 sample), “possible” case (at least one level 2 but no level 3 samples), and “uncertain” case (at least one level 1 but no level 2 or 3 samples).

Overall, two probable and ten possible tularemia case animals were considered infected with *F. tularensis*, including 7/36 (19.44%) Cape hares and 5/38 (13.15%) wild rabbits (Table 1). The uncertain cases corresponded to 8/36 (22.22%) Cape hares and 11/38 (28.94%) wild rabbits. The geographic distribution of probable and possible cases included the provinces Tipaza (5 hares and 5 wild rabbits), Medea (1 hare), and Ain El Defla (1 hare)). These animals included 7 males and 5 females, and 11 mature animals and 1 juvenile. They were collected in March (n = 1), August (2), September (1), October (2), November (4), and December (2). The uncertain cases were detected in Tipaza (7 hares and 9 wild rabbits), Blida (2 wild rabbits), and Algiers (1 hare). These animals included 12 males, 7 females, 16 mature animals, and 3 juveniles. They were collected in January (1), March (4), April (1), July (1), August (1), October (3), November (2), and December (6).

### Pathological findings

**Figure 2:**
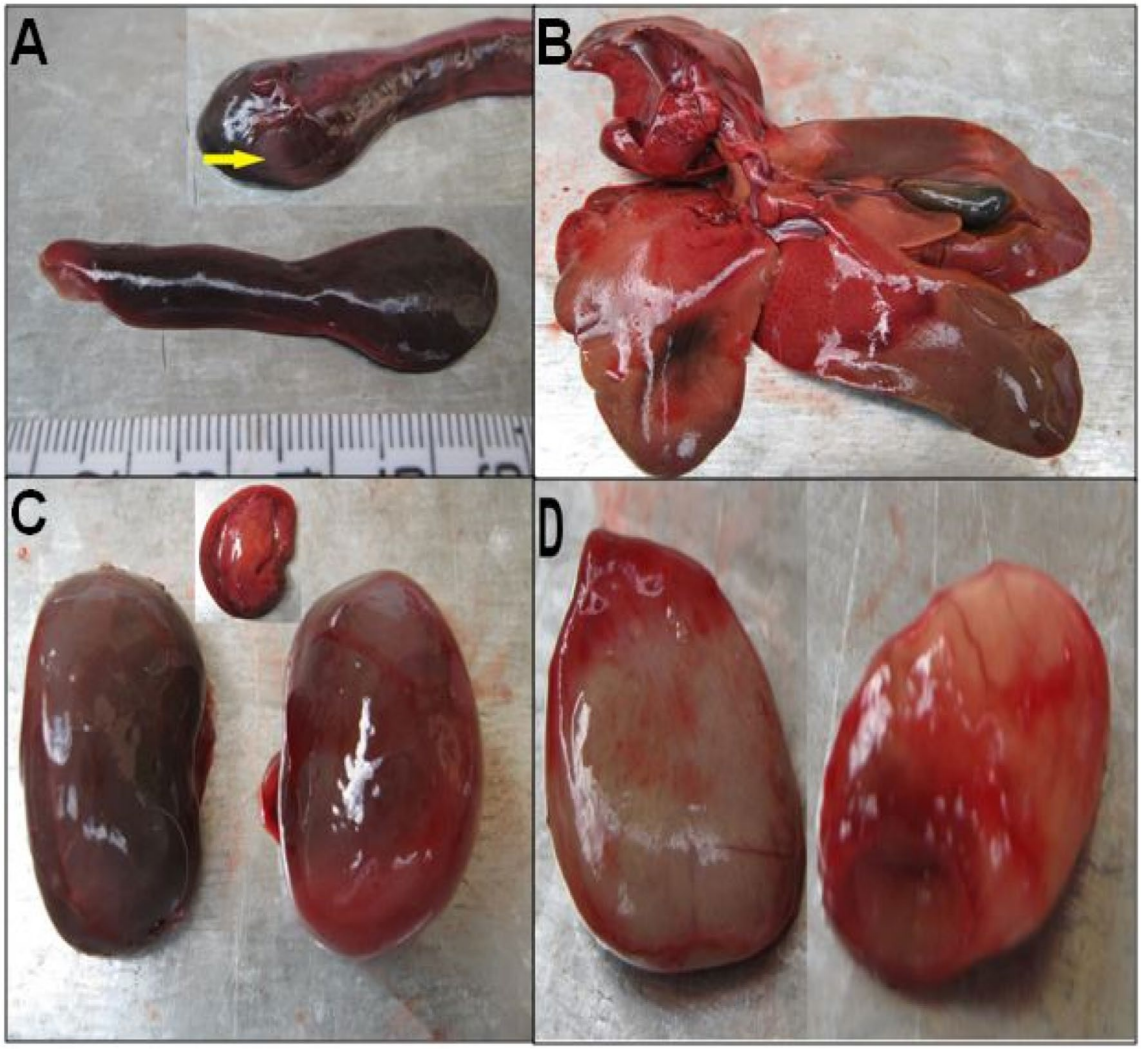
Organ lesions in Cape hare (cadaver). (**A**): Splenomegaly. The spleen is markedly enlarged (6 cm in length) and has prominent rounded edges with some discoloration areas. (**B**): Heterogeneous discoloration in the liver lobes with hemorrhage areas and an enlarged and full gallbladder. (**C**): Congestion of the kidney with capsule congestion. (**D**): The enlargement of adrenal glands.

**Figure 3:**
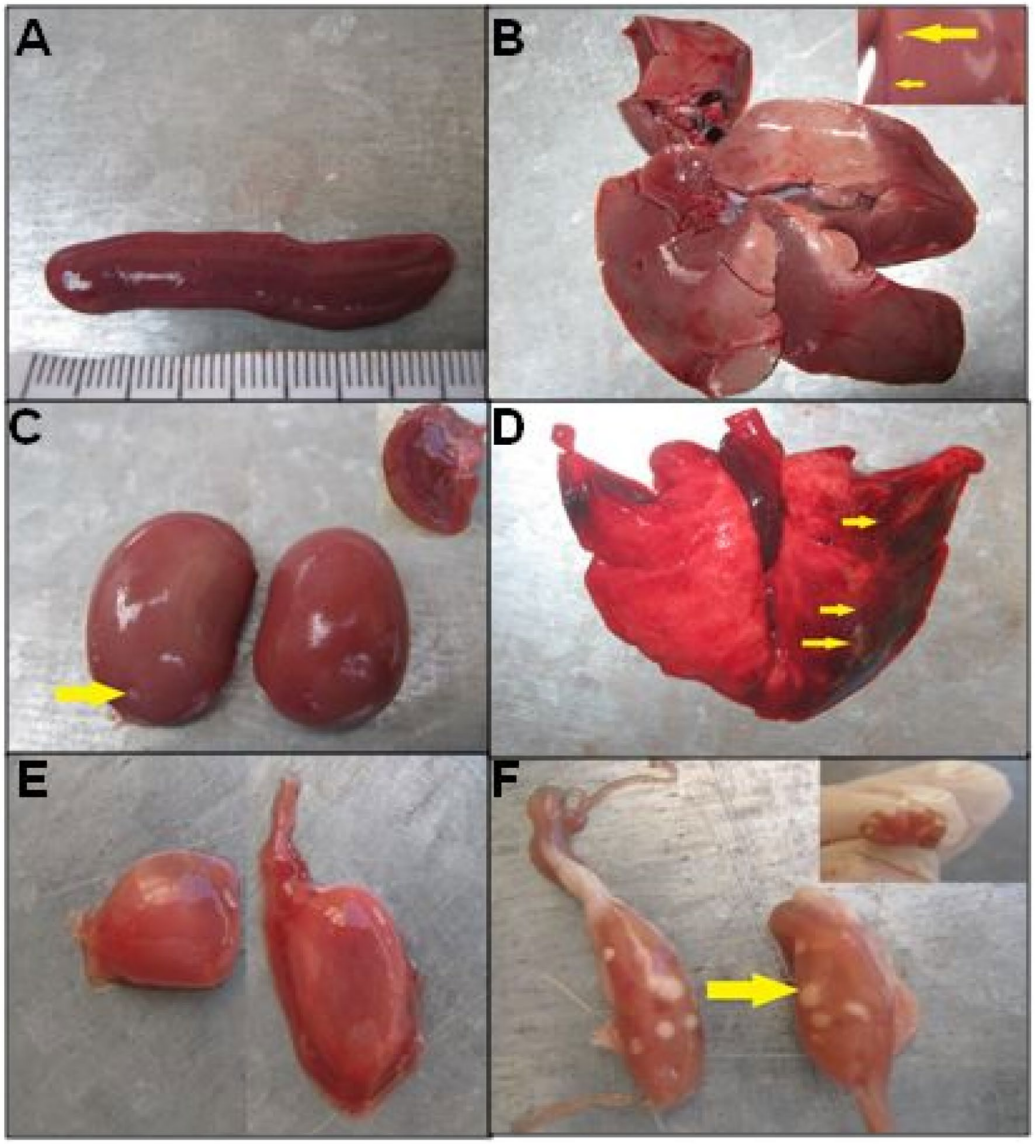
Organ lesions in wild rabbit (Hunted animal). (**A**): Splenomegaly. The spleen is markedly enlarged (5 cm in length) and has prominent rounded edges. (**B**): Heterogeneous discoloration in the liver lobes with hemorrhage areas and a few small white necrotic foci (arrow). (**C**): Congestion of the kidney with a white focus of necrosis foci in the capsule (arrow). (**D**): Pneumonia with multifocal hemorrhages and dark hyperemic areas are scattered throughout the lungs, also few small white necrotic foci (arrow). (**E**): The enlargement of adrenal glands. (**F**): Numerous necrotic white foci in the ovary.

Of the 12 probable and possible tularemia cases, only one hare was found dead, while 11 animals were hunted (the two probable cases were hunted animals). The body condition of these 12 animals was good. Two were found dead for the 19 uncertain cases (one from a traffic accident), while 17 were hunted. The body condition of the 19 uncertain cases was good in 18 cases and weak for one hunted rabbit. Of the 43 negative leporids, most (i.e., 20 hares and 20 wild rabbits) were in good body condition, and only one dead hare and two hunted wild rabbits were in weak condition. The main pathological findings were retrieved from the hares and wild rabbits diagnosed with probable or possible tularemia. Details of organ lesions detected at necropsy in all collected animals are presented in Tables S2 and S3. Figures 2 and 3 show the main lesions observed in tularemic animals. In addition, Table S4 indicates the degree of organ involvement (severe or moderate).

### Immunohistochemistry (IHC) examination

**Figure 4:**
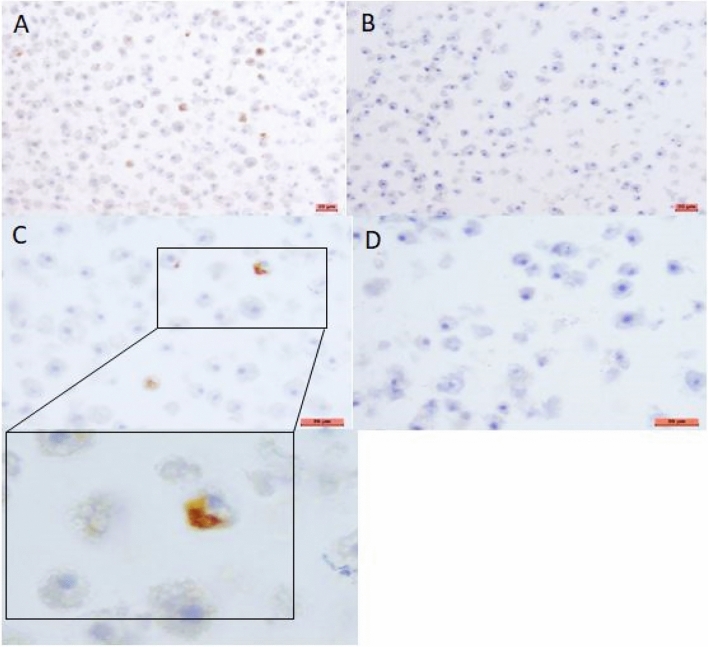

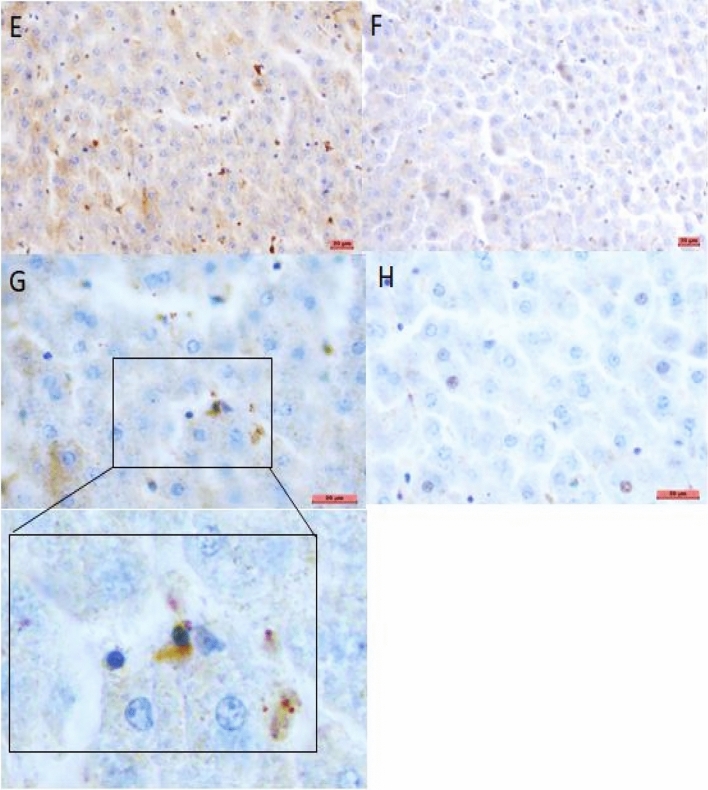
Immunohistochemistry results. (**A**) and (**C**) positive control corresponding to *Francisella tularensis* in amoebae labeled with FB11 monoclonal antibody 1/1000 in Leica dilution buffer. (**B**) and (**D**) negative control corresponding to *Francisella philomiragia* in amoebae labeled with FB11 monoclonal antibody. (**E**) and (**G**) show *Francisella tularensis* labeled in the cytosol of liver cells. (**F**) and (**H**) show the background generated by the Leica revelation system without antibodies. A, B, E and F: objective × 20. C, D, G, and H: objective × 40. Added insert: objective × 100.

IHC was conducted on four specimens graded level 3 (two spleens and two livers) from two animals. It was also performed on four specimens graded level 2 (two spleens and two livers) from two other animals. IHC was negative for all the spleen samples. In contrast, for one level 2 liver sample, bacteria were revealed by the anti-*F. tularensis* antibody within the hepatocytes' cytosol (Fig. 4).

### Ectoparasites

Potential arthropod vectors of tularemia were found for two probable tularemia cases. One hare was infested by nymphs of *Rhipicephalus *spp*.* and *Hyalomma *spp*.* One wild rabbit was infested by *Ixodes ricinus* and *Spilopsyllus cuniculi*. Concerning the ten possible cases, five animals were infested by known tularemia vectors (4 cases by *I. ricinus* and one case each by *Rh. sanguineus*, *S. cuniculi*, or lice). In addition, three animals were infested by *Rhipicephalus *spp, *Hyalomma *spp*,* and fleas (*Ctenocephalides canis* and *Ctenocephalides felis*). Finally, 13 of the uncertain cases were infested by tularemia vectors (*I. ricinus*, nine cases; *S. cuniculi,* five cases; *Rh. sanguineus*, one case; and *Haemodipsus lepori* and *Haemodipsus setoni,* one case). Other ectoparasites were detected, including *Rhipicephalus pusilus*, *Rhipicephalus *spp, *Hyalomma *spp, *C. felis, C. canis,* and mites. The qPCR-negative animals were also infested by some tularemia vectors (*I. ricinus,* 11 cases; *S. cuniculi,* 5 cases; and *Rh. sanguineus,* 3 cases) (Table S5).

## Discussion

Tularemia affects animal welfare, human health, and the environment and is thus better approached from a one-health perspective^27^. Several studies in the Northern hemisphere^28^, and more recently in Australia^15,16^, have provided a vital research track in the epidemiology of this disease. In contrast, studies in Africa are too limited and scarce. The aim of this study was to investigate the presence of tularemia in wild leporids collected in Northern Algeria. These animals are highly susceptible to *F. tularensis* infection and considered sentinel hosts for surveillance of tularemia. The strategy we used to detect *F. tularensis* in leporids mainly used molecular, histological and immunohistochemical analyzes of tissues taken from animals found dead or hunted. To the best of our knowledge, detection of *F. tularensis* by PCR or culture has not been previously reported in wild leporidae in Algeria or other African countries.

Animal tissue samples were tested using three qPCR assays of variable sensitivity and specificity. The Type B-qPCR test targets a specific junction between IS*Ftu2* and a flanking 3′ region, which is considered specific for *F. tularensis* subsp. *holarctica*^26^, the only tularemia agent found in Europe and Asia. The Tul4-qPCR assay targets a simple copy gene encoding a surface protein, which can be found in the genome of all *F. tularensis* subspecies causing tularemia and that of the aquatic bacterium *F. novicida*. Because *F. novicida* has never been isolated from lagomorphs or other animal species, and very rarely from human^29^, a positive Tul4 qPCR for the studied tissue samples likely indicated the presence of *F. tularensis* DNA. The IS*Ftu2* qPCR is considered highly sensitive because multiple copies of this insertion sequence are found in the *F. tularensis* genome. However, it lacks specificity because IS*Ftu2* is also found in many other *Francisella* species^25^.

Two animals were considered “probable” tularemia cases because some of their samples were positive for the three qPCR tests. Ten animals were considered “possible” tularemia cases because their samples were positive for the ISFtu2 and Tul4 qPCRs but not the Type B qPCR. Finally 19 leporids were “uncertain” cases because only samples positive for the ISFtu2 qPCR were found. For the remaining 43 animals, all the tested samples were negative for the three qPCRs. Overall, we detected *F. tularensis* DNA-positive samples in 12/74 (16.21%) leporids, which strongly suggest that tularemia is present in the lagomorph population of the study area. The positive Type B qPCR tests in two animals suggested that *F. tularensis* subsp. *holarctica* could be the involved subspecies. We did not confirm these data by isolating *F. tularensis* from the studied leporids. However, the isolation of this pathogen from human or animal samples is tedious and has a low sensitivity^13^. Moreover, most of our samples were not appropriate for *F. tularensis* culture because of their long-term preservation in ethanol 70° or 10% formalin. Further study using fresh (non-fixed) tissue samples from dead leporids collected in the same study area is needed to definitively confirm the presence of tularemia in these animals and characterize the *F. tularensis* subspecies and genotypes involved.

Although PCR is usually more sensitive than culture for detecting *F. tularensis*, it also has some limitations. Firstly, the DNA extraction from organs preserved in ethanol for several months was difficult although easier for spleen than for liver samples. Some tissue samples could be lysed only after overnight incubation with proteinase K. Secondly, tissue samples contained PCR inhibitors as demonstrated by better DNA amplification from some samples after their dilution in PCR grade water. To reduce the effect of PCR inhibitors, organ samples with negative qPCR were retested using Bovine Serum Albumin (BSA) and the Real-time PCR system TaqMan (Applied Biosystems, Munich, Germany)^30^. Finally, DNA regions to be amplified were optimized to obtain high sensitivity and specificity of qPCR tests.

IHC detection of *F. tularensis* in formalin-fixed tissue can be helpful for tularemia diagnosis^31,32^. For one possible tularemia case, *F. tularensis* could be detected on immunohistochemical (IHC) examination of a liver sample using a specific anti-*F. tularensis* antibody. The intensity and localization of positive staining were comparable to those previously recorded for other animals^32,33^. IHC did not provide interpretable findings for four other tested specimens. Such negative results might be explained by an inhomogeneous distribution of infectious foci in the involved organs as well as a low bacterial inoculum in infected tissues. This has been previously demonstrated in tularemia granulomatous lesions in cell types like epithelial cells of the kidney, testis, and epididymis, hepatocytes, and bronchiolar epithelial cells^31^. Besides, IHC is a delicate technology whose results are highly dependent on the quality and fixation time of the organ tissues^34^. IHC analysis of dead animal tissues remains challenging, especially in case of tissue necrosis^34^.

In our limited case series we found a *F. tularensis* infection prevalence in leporids of 2.7% (2/74) for probable tularemia cases and 16.2% (12/74) when considering both probable and posible cases. We cannot make a guess about the prevalence of tularemia because our series is not representative of the general lagomorph population in the study area. In Germany, *F. tularensis* DNA was detected in 1.1% of European Brown hares and 2.4% of wild rabbits collected between 2009 and 2014^35^. Higher infection rates were reported in the same country, including 11.8% (100/848 animals) in hares collcted in the North Rhine-Westphalia region^36^ and 30% (55/179) in brown hares collected between 2010 and 2016 in Baden-Wuerttemberg^37^. In Hungary, the prevalence of tularemia in hares was evaluated at 4.9–5.3%^38^. In Portugal, prevalences of 4.3% and 6.3% were reported in brown hares and wild rabbits, respectively^39^. However, the comparison of the reported tularemia prevalences in leporids is irrelevant because studies involved different animal species and geographic areas, and used different methods for *F. tularensis* detection.

Two possibilities could explain the lack of detection of tularemia in Algeria before this study. The first hypothesis is that this disease was not searched for in previous years, while it could have been present in this country for decades. The second hypothesis is that tularemia was recently imported in Algeria. Migratory birds may have been involved in the long-distance spread of *F. tularensis*^40^. These hosts can be infested by ectoparasites such as ticks which are the primary vectors of tularemia^41,42^. They can also spread the bacteria in the hydro-telluric environment through their secretions and feces^18,43,44^. An alternative possibility is that *F. tularensis*-infected animals (especially game animals) have been imported in Algeria from endemic countries. Whatever the mode of introduction of tularemia in Algeria, the dissemination of this disease over time might have been facilitated by the ability of *F. tularensis* to infect multiple hosts and its better survival in a cool environment^45^, which characterizes Northern Algeria climate. The emergence or re-emergence of tularemia in other countries has been related to climate change, human-mediated movement of infected animals, and wartime resulting in a significant rise of *F. tularensis* infections in the rodent populations^39,46^.

In our study, infected animals were collected throughout 4 years, although more frequently in autumn. Probable and possible tularemia cases were mainly collected during the hunting season (i.e., September, October, November, and December). Animals could not be collected in February because of heavy rains and in May and June because it corresponds to female leporids' lactation period. In most endemic countries, tularemia cases are typically more frequent in late spring, the summer months, and early autumn^37,47–50^. Occasionally, fatal tularemia cases in hares have been predominantly reported during the cold season^11,51^. The climatic conditions can affect tularemia outbreaks in animals, depending on the reservoir involved and the predominant modes of infection^52^.

We detected tularemia more frequently in female than in male hares, and the reverse was true for wild rabbits. The prevalence of tularemia in male or female lagomorphs varies between studies. In Sweden, Morener et al.^50^ reported a tularemia case series only involving male hares. In the same country, Borg et al.^50^ observed an overrepresentation of females in the epizootic of 1967. They suggested that, compared to males, females had a higher risk of exposure to infected mosquitoes or were more vulnerable to tularemia because they were pregnant or had just given birth to a litter^50^. Tularemia was found in a few juveline leporids, which might be explained by a shorter exposure time to *F. tularensis*, a higher death rates due to higher susceptibility to *F. tularensis* infection or easier predation by their natural enemies, or more frequent hunting of adults compared to the juveniles^53^.

Tularemia is usually more frequently detected in leporids found dead than in hunted animals. As an example, a German study reported a higher prevalence of tularemia in hares found dead (2.9%) than in hunted ones (0.7%)^35^. In our study, most qPCR-positive animals were hunted. Our study might not be representative of the prevalence of tularemia in either population because most collected animals had been hunted.

The incubation period and clinical presentation of tularemia in leporids vary according to the species considered. Tularemia is typically an acute disease in mountain hares (*Lepus timidus*) in Scandinavia and has a chronic pattern in European brown hares (*Lepus europaeus*) in Central Europe^50^. The incubation time and clinical presentation of tularemia can be different in Cape hares (*Lepus capensis*). Wild rabbits are less sensitive to *F. tularensis* infection than hares^31,39,54^. An extended incubation period and chronic evolution of tularemia would facilitate the detection of *F. tularensis* in infected animals. In our study, a similar tularemia prevalence was found in the Cape hares and wild rabbits, which might reflect exposure to a same biotope area and environmental reservoirs of *F. tularensis*.

The pathological lesions of tularemiia in leporids can vary according to the *F. tularensis* strain involved, the mode and route of infection, and the susceptibility and immune status of the host^32,50^. In the European brown hares, granulomas with central necrosis have been reported in the lungs and kidneys and occasionally in the liver, spleen, bone marrow, and lymph nodes^50^. In contrast, only acute necrosis in the liver, spleen, bone marrow, and lymph nodes have been found in *Lepus timudus* hares in Sweden^50^. The lesions in the Japanese hare (*Lepus brachyurus angustidens*) are comparable to those of *Lepus timidus*, except for cutaneous, lung, brain, and adrenal gland lesions^32^. In the European rabbit, *Oryctolagus cuniculus*, tularemia is not associated with identifiable macroscopic tissue lesions^39,55^. To our knowledge, no reports describing post-mortem lesions in Cape hares with tularemia are available. In this study, similar lesions were found in hares and wild rabbits except necrotic foci only observed in some wild rabbit organs (such as liver, lungs, kidney, ovary). Most animals had pathological lesions of pneumonia, gastritis and enteritis. Kidney lesions and adrenal glands enlargment were oberved. Necrotic lesions were occasionally found in the lungs, liver, spleen and ovary and hemorrhages in the lungs, liver, and intestines.

Tularemia is an arthropod-born disease in most endemic areas^14,22,28^. In our study, 50% of positive leporids were infested by known tularemia vectors such as ticks (*Ixodes ricinus*^56,57^, *Rhipicephalus sanguineus*^39^*)*, fleas (*Spillopsylus cuniculi*^58^*)*, and lice of lagomorphs (*Haemodipsus lepori* and *Haemodipsus setoni*^59,60^). Ticks are the most significant arthropod vectors of tularemia^61^. Ticks are frequently involved in the transmission of tularemia in North America, including *Dermacentor andersoni*, *D*. *variabilis*, and *Amblyomma americanum*^57,62,63^*.* In Europe, tick-borne tularemia represents 13% to 26% of human cases^57,64^. The involved species include *D. marginatus*, *D. reticulatus*, *I. ricinus, R. sanguineus,* and *Haemaphysalis concinna*^65,66^*.* Further research on wild leporid sucking arthropods is needed to confirm the presence and clarify the ecology of *F. tularensis* in Algeria.

Our study reports for the first time the detection of *F. tularensis* DNA in leporids from Northern Algeria. The markers most in favor of tularemia in the animals studied are the positivity of qPCR tests, in particular, the "type B" qPCR test which amplifies a specific DNA sequence of *F. tularensis* subsp. *holarctica*, and a positive immunohistological examination in one animal. Further investigation is needed to confirm our results by the isolation of this pathogen from animal samples and determine the *F. tularensis* subspecies and genotypes involved. This would allow the characterization of the *F. tularensis* subspecies and genotypes present in Algeria. Furthermore, our findings push us in future studies to seek tularemia in the Algerian human population. To achieve this, interdisciplinary or trans-disciplinary collaborative efforts underpinned by the One Health concept will be necessary.

## Materials and methods

### Animal samples

During the period 2014–2016 and in 2018, we collected 74 wild leporids (36 Cape hares and 38 wild rabbits) shot by hunters (n = 60) or found dead (n = 14) for post-mortem examination. A questionnaire was completed for each animal with information on the age (adult/juveniles), sex, region of collection, and the hunter or person who discovered the animal. Tissue samples (spleen, liver) were collected and stored in ethanol 70° for PCR testing or 10% buffered formalin for histology and immunohistochemistry analyses.

### Necropsy examination of animals

The skin was scanned for the presence of ectoparasites or the lesions they cause. The following lesions were sought in the organs: enlargement, hemorrhagic lesions, congestion, edema, necrosis, degeneration, and inflammation. These lesions were graded as mild, moderate, or severe.

### Detection of *Franscisella tularensis* DNA

#### DNA extraction from animal tissues

A tissue sample of 25 mg was lysed by incubation in proteinase K overnight at 56 °C. Then, the DNA was extracted using the NucleoSpin Blood kit (Macherey Nagel, Hoerdt, France) according to the manufacturer's instructions.

#### Real-time PCR (qPCR)

*Francisella tularensis* DNA was detected in animal samples using three real-time PCR (qPCR) tests. The ISFtu2-qPCR targets the IS*Ftu*2 insertion sequence, which is present in multi-copy in the genomes of all *Francisella* species and *Francisella*-like endosymbionts of arthropods. The Tul4-qPCR targets the gene encoding Tul4, a membrane protein of 17 kDa found in all subspecies of *F. tularensis* and the aquatic species *F. novicida*^25^. The Type B-qPCR targets a specific junction between ISFtu2 and a flanking 3' region, only found in *F tularensis* subsp. *holarctica*, the only subspecies causing tularemia in Eurasia^26^. The TaqMan Fast Advanced PCR Master Mix kit (ThermoFisher Scientific Walthman, US) was used. The qPCR mixes also contained 100 ng of DNA extract, 10 µM of primers, and 2 µM of the appropriate probe (Table S1). When PCR tests were negative, they were repeated with 0.4 µL BSA (1 ng/µL) to block the potential PCR inhibitors. qPCR tests were run on the Lightcycler 480 (Roche Diagnostics, Meylan, France), and the PCR program was 50 °C for 2 min, 95 °C for 2 min, and 45 cycles at 95 °C for 3 s and 60 °C for 30 s. All qPCR tests were performed in duplicate or triplicate for inconsistent results. A qPCR test was considered positive only when two replicate tests were positive and using a cycle threshold (Ct) value < 38 for ISFtu2-qPCR and < 40 for Tul4-qPCR and Type B-qPCR. The specimens were classified into four categories according to qPCR tests results: level 3 for a sample positive for the three qPCR tests; level 2 when only ISFtu2- and Tul4-qPCR were positive; level one when only ISFtu2-qPCR was positive, and negative when all qPCR tests were negative.

The qPCR controls were performed with DNA extracts from the following strains: *F. philomiragia* ATCC 25,015, *F. noatunensis* LMG 23,800, *F. tularensis* subsp. *novicida* U112, and the clinical strain of *F. tularensis* subsp. *holarctica*. Our *F. tularensis* collection has been approved by the Agence Nationale de Securité du Médicament et des Produits de santé (France) (ANSM, authorization number ADE-103892019-7).

#### Immunohistochemistry analysis

Immunohistochemistry (IHC) analysis was performed for qPCR-positive spleen and liver samples from four animals. These samples were classified as level 3 (n = 4, two spleens, two livers) or level 2 (n = 4, two spleens, and two livers). IHC was performed on 3 µm paraffin sections using Bond Polymer Refine Detection (#DS9800, Leica Biosystems, Newcastle, UK).

Deparaffinized slides were incubated in a citrate solution (pH = 6) for 20 min, at 100 °C for epitopes retrieval. Staining was performed using an *F. tularensis* LPS-directed mouse monoclonal antibody (FB11, used at 1:1000, ThermoFisher Scientific, Rockford USA) applied for one hour at room temperature. Peroxidase revelation was performed using the Bond Polymer Refine Detection Kit (Leica Biosystems, Newcastle, UK), according to the manufacturer's recommendations. The pictures were taken with a Leica DFC295 camera, using the Leica Application Suite 4.13.

The positive control was *F. tularensis-*infected amoebae fixed with 4% formaldehyde assembled in a cytobloc using Shandon reagent and paraffin-embedded. The negative control was *Francisella philomiragia-*infected amoebae treated at the same time and similarly to the positive control.

### Tularemia case definition

According to the specificities of the qPCR assays for *F. tularensis* detection, the animals were considered as a: 1/probable tularemia case when at least one sample was level 3; 2/possible tularemia case when at least one sample was level 2 (but no sample was level 3); 3/uncertain tularemia case when at least one sample was level 1 (but no sample as level 3 or level 2); and 4/not infected by *F. tularensis* when all three qPCR were negative for all tested samples. Pathological and immunohistochemistry findings could not be used in this definition because the former lacked specificity, and the latter was only performed on a few samples.

### Ethical statement

All animal experiments were approved by the Algerian ethics committee of the General Directorate of Forests of the Ministry of Agriculture, Rural Development and Fisheries (GDF-MARDF). All methods were carried out in accordance with relevant guidelines and regulations, and in accordance with ARRIVE guidelines. The animal corpses were obtained during legal hunting activities in accordance with the laws of the Algerian Republic and the DGF-MARDF.

## Supplementary Information

Supplementary Tables.
