# Supplementary material for: Francisella tularensis PCR detection in Cape hares (Lepus capensis) and wild rabbits (Oryctolagus cuniculus) in Algeria
Source: Sci Rep. 2022 Dec 12;12:21451. doi: 10.1038/s41598-022-25188-0 (PMC9743112; doi:10.1038/s41598-022-25188-0)
Supplement: Supplementary file 1 — Supplementary Tables. [file 41598_2022_25188_MOESM1_ESM.docx]

***Francisella tularensis* PCR detection in Cape hares (*Lepus capensis*)**

**and wild rabbits (*Oryctolagus cuniculus*) in Algeria**

**Imene Ammam^1, 2^, Camille D. Brunet ^3^, Nouria Boukenaoui-Ferrouk^1, 4^, Julien Peyroux^3^, Sylvie Berthier^5^, Jean Boutonnat^5^, Karim Rahal^1^, Idir Bitam^2, 6^, Max Maurin^7^*.**

### Author Affiliations

**^1:^** Institute of Veterinary Sciences, University of Blida 1, Algeria.

**^2:^** Laboratory of Biodiversity and Environment: Interactions, Genome, University of Sciences and Technology Houari Boumedienne, Algiers, Algeria.

**^3^**: Grenoble Alpes University, CNRS, TIMC UMR5525, 38000 Grenoble, France.

**^4^:** Houari Boumediene University of Science and Technology (USTHB), Faculty of Biological Sciences (FSB), Laboratory of Research on Arid Zones Lands (LRZA), Bab Ezzouar, BP 32, 16111 Algiers, Algeria.

^5^: Grenoble Alpes University Hospital Center, 38000 Grenoble, France; Centre Hospitalier Universitaire Grenoble Alpes, Grenoble, France.

**^6:^** Superior School of Food Sciences and Food Industries of Algiers, Algeria.

**^7^:** Grenoble Alpes University, CNRS, Grenoble INP, CHU Grenoble Alpes, TIMC UMR5525, 38000 Grenoble, France.

***Corresponding author :**

Max Maurin; CHU Grenoble Alpes, University Grenoble Alpes, Bd Chantourne, 38043 Grenoble, France. Mail: [mmaurin@chu-grenoble.fr](mailto:mmaurin@chu-grenoble.fr) .

**Table S1.** Primers and probes used for detection of *F. tularensis.*

| **ISFtu2** | Primer (Fw) | ttggtagatcagttggtgggataac |
| --- | --- | --- |
|  | Primer (Rw) | tgagttttaccttctgacaacaatatttc |
|  | Probe | aaatccatgctatgactgatgctttaggtaatcca |
| **Tul4** | Primer (Fw) | attacaatggcaggctccaga |
|  | Primer (Rw) | tgcccaagttttatcgttcttct |
|  | Probe | ttctaagtgccatgatacaagcttcccaattactaag |
| **Type B** | Primer (Fw) | cttgtacttttatttggctactgagaaact |
|  | Primer (Rw) | cttgcttggtttgtaaatatagtggaa |
|  | Probe | acctagttcaacctcaagacttttagtaatgggaatgtca |

**Table S2.** Number of organs with lesions in Hares and wild rabbits that were positives and suspects to be infected with *F.tularensis*

| **Organs** | **Presence** | | | | | | **Absence** | | | | | | **Not Determined^*^** | | | | | |
| --- | --- | --- | --- | --- | --- | --- | --- | --- | --- | --- | --- | --- | --- | --- | --- | --- | --- | --- |
|  | **Hare** | | | **Wild rabbit** | | | **Hare** | | | **Wild rabbit** | | | **Hare** | | | **Wild rabbit** | | |
| **Disease level*** | **Level 3** | **Level 2** | **Level 1** | **Level 3** | **Level 2** | **Level 1** | **Level 3** | **Level 2** | **Level 1** | **Level 3** | **Level 2** | **Level 1** | **Level 3** | **Level 2** | **Level 1** | **Level 3** | **Level 2** | **Level 1** |
| **Spleen** | 1/1 | 6/6 | 8/8 | 1/1 | 4/4 | 11/11 | 0/1 | 0/6 | 0/8 | 0/1 | 0/4 | 0/11 | 0/1 | 0/6 | 0/8 | 0/1 | 0/4 | 0/11 |
| **Liver** | 1/1 | 5/6 | 7/8 | 1/1 | 4/4 | 10/11 | 0/1 | 0/6 | 0/8 | 0/1 | 0/4 | 0/11 | 0/1 | 1/6 | 1/8 | 0/1 | 0/4 | 1/11 |
| **Lungs** | 1/1 | 5/6 | 7/8 | 1/1 | 2/4 | 10/11 | 0/1 | 0/6 | 0/8 | 0/1 | 0/4 | 0/11 | 0/1 | 1/6 | 1/8 | 0/1 | 0/4 | 1/11 |
| **Kidney** | 1/1 | 6/6 | 8/8 | 1/1 | 4/4 | 11/11 | 0/1 | 0/6 | 0/8 | 0/1 | 0/4 | 0/11 | 0/1 | 0/6 | 0/8 | 0/1 | 0/4 | 0/11 |
| **Adrenal gland** | 1/1 | 6/6 | 7/8 | 1/1 | 3/4 | 11/11 | 0/1 | 0/6 | 1/8 | 0/1 | 0/4 | 0/11 | 0/1 | 0/6 | 0/8 | 0/1 | 1/4 | 0/11 |
| **Stomach** | 1/1 | 5/6 | 8/8 | 1/1 | 2/4 | 8/11 | 0/1 | 0/6 | 0/8 | 0/1 | 2/4 | 0/11 | 0/1 | 1/6 | 0/8 | 0/1 | 0/4 | 2/11 |
| **Intestine** | 1/1 | 5/6 | 8/8 | 1/1 | 3/4 | 8/11 | 0/1 | 0/6 | 0/8 | 0/1 | 1/4 | 0/11 | 0/1 | 1/6 | 0/8 | 0/1 | 0/4 | 2/11 |

* levels 1, 2, and 3 correspond respectively to probable, possible, and uncertain tularemia cases

**Table S3**. Number of animals (n/tested) with pathological lesions in different organs according to level of infection.

| **Organ** | **Pathological lesions** | **Level 3*** | | **Level 2*** | | **Level 1*** | |
| --- | --- | --- | --- | --- | --- | --- | --- |
|  |  | **Hares** | **Wild rabbits** | **Hares** | **Wild rabbits** | **Hares** | **Wild rabbits** |
| Spleen | Enlargement  Discoloration  Necrose | 1/1  0/1  0/1 | 1/1  0/1  0/1 | 6/6  3/6  0/6 | 4/4  1/4  ¼ | 8/8  0/8  0/8 | 11/11  7/11  0/11 |
| Liver | Discoloration  Hemorrhage  Necrose  Friable | 1/1  1/1  0/1  0/1 | 1/1  0/1  0/1  1/1 | 5/5  4/5  0/5  1/5 | 4/4  4/4  3/4  0/4 | 6/7  7/7  0/7  0/7 | 8/10  10/10  4/10  2/10 |
| Lung | Pneumonia  Hemorrhage  Necrose  Hyperhemic areas | 1/1  1/1  0/1  1/1 | 1/1  1/1  0/1  1/1 | 5/6  5/6  0/6  6/6 | 4/4  4/4  0/4  2/4 | 6/7  4/7  0/7  3/7 | 9/10  7/10  2/10  8/10 |
| Kidney | Cortex congestion  Pelevis congestion  Capsule lesion^$^a | 1/1  1/1  0/1 | 1/1  1/1  0/1 | 6/6  5/6  1/6 | 4/4  3/4  1/4 | 8/8  7/8  0/8 | 11/11  8/11  2/11 |
| Adernal gland | Enlargement | 1/1 | 1/1 | 6/6 | 3/3 | 7/8 | 11/11 |
| Stomach | Gastritis | 1/1 | 1/1 | 5/5 | 2/4 | 8/8 | 6/9 |
| Intestine | Enteritis  Hemorrhage  Vessel dilatation | 1/1  0/1  0/1 | 1/1  0/1  0/1 | 5/5  2/5  3/5 | 3/4  0/4  4/4 | 8/8  4/8  4/8 | 7/9  2/9  5/9 |

^£^: Other lesions not listed in the table comprised necrotic foci in an ovary (n=1) in a wild rabbit belonging to the level 1 of disease.

^$:^ Capsule lesion : 1 case (hare) with congestion , 1 case ( wild rabbit) with necrotic foci.

* levels 1, 2, and 3 correspond respectively to probable, possible, and uncertain tularemia cases

**Table S4.** Number of animals (n/tested) with severe or moderate lesions in different organs according to level of infection.

| **Infection** | **Level 3*** | | | | **Level 2*** | | | | **Level 1*** | | | |
| --- | --- | --- | --- | --- | --- | --- | --- | --- | --- | --- | --- | --- |
| **Animals** | **Hare** | | **Wild rabbit** | | **Hare** | | **Wild rabbit** | | **Hare** | | **Wild rabbit** | |
| **Lesion** | **Severe** | **Moderate** | **Severe** | **Moderate** | **Severe** | **Moderate** | **Severe** | **Moderate** | **Severe** | **Moderate** | **Severe** | **Moderate** |
| **Spleen** | 1/1 | 0/1 | 1/1 | 0/1 | 5/6 | 1/6 | 3/4 | 1/4 | 1/8 | 7/8 | 7/11 | 4/11 |
| **Liver** | 1/1 | 0/1 | 1/1 | 0/1 | 5/5 | 0/5 | 3/4 | 1/4 | 2/7 | 5/7 | 7/10 | 3/10 |
| **Lungs** | 1/1 | 0/1 | 1/1 | 0/1 | 4/5 | 1/5 | 2/4 | 2/4 | 5/7 | 2/7 | 6/10 | 4/10 |
| **Kideny** | 1/1 | 0/1 | 1/1 | 0/1 | 5/6 | 1/6 | 3/4 | 1/4 | 1/8 | 7/8 | 5/11 | 6/11 |
| **Adrenal**  **gland** | 1/1 | 0/1 | 1/1 | 0/1 | 5/6 | 1/6 | 3/3 | 0/3 | 4/7 | 3/7 | 10/11 | 1/11 |
| **Stomach** | 0/1 | 1/1 | 0/1 | 1/1 | 1/5 | 4/5 | 0/2 | 2/2 | 4/8 | 4/8 | 2/8 | 6/8 |
| **Intestine** | 0/1 | 1/1 | 0/1 | 1/1 | 1/5 | 4/5 | 1/3 | 2/3 | 4/8 | 4/8 | 3/9 | 6/9 |

* levels 1, 2, and 3 correspond respectively to probable, possible, and uncertain tularemia cases

**Table S5**. Number of animals (n/tested) with ectoparasites (known as tularemia vectors or not) infesting lagmorphs according to the level of infection.

| **Disease Level** | **Level 3** | | **Level 2** | | **Level 1** | | **Level 0** | |
| --- | --- | --- | --- | --- | --- | --- | --- | --- |
| **Animals** | **Hare** | **Wild**  **rabbit** | **Hare** | **Wild**  **rabbit** | **Hare** | **Wild**  **rabbit** | **Hare** | **Wild**  **rabbit** |
| **All ectoparasites** | 1/1 | 1/1 | 5/6 | 2/4 | 6/8 | 10/11 | 14/21 | 12/22 |
| **Tularemia vectors** | 0/1 | 1/1 | 3/6 | 1/4 | 4/8 | 10/11 | 7/21 | 9/22 |
| **Non-tularemia vectors** | 1/1 | 0/1 | 2/6 | 1/4 | 4/8 | 4/11 | 10/21 | 9/22 |

* levels 1, 2, and 3 correspond respectively to probable, possible, and uncertain tularemia cases

**Table S6.** Number of animals (n/tested) not infected with F. tularensis (all qPCR tests negative) with organ lesions

| **Organ lesions** | **Presence** | | **Absence** | | **Not Determined** | |
| --- | --- | --- | --- | --- | --- | --- |
|  | **Hare** | **Wild rabbit** | **Hare** | **Wild rabbit** | **Hare** | **Wild rabbit** |
| **Spleen** | 20/21 | 20/22 | 1/21 | 0/22 | 0/21 | 2/22 |
| **Liver** | 20/21 | 21/22 | 1/21 | 0/22 | 0/21 | 1/22 |
| **Lungs** | 21/21 | 21/22 | 0/21 | 0/22 | 0/21 | 1/22 |
| **Kidney** | 19/21 | 18/22 | 2/21 | 1/22 | 0/21 | 3/22 |
| **Adrenal gland** | 19/21 | 20/22 | 0/21 | 0/22 | 2/21 | 2/22 |
| **Stomach** | 17/21 | 14/22 | 4/21 | 5/22 | 0/21 | 3/22 |
| **Intestine** | 19/21 | 18/22 | 1/21 | 1/22 | 1/21 | 3/22 |

**Table S7.** QPCR Ct values for *Francisella tularensis* qPCR tests.

| **Samples** | | | **Isftu2** | | | **Tul4** | | | **Type B** | | | **Level of**  **infection*** | |
| --- | --- | --- | --- | --- | --- | --- | --- | --- | --- | --- | --- | --- | --- |
| **Animals** | **Organ** | **test 1** | | **test 2** | **test 1** | | **test 2** | **test 1** | | **test 2** |  | |  |
| Hare 1 | Spleen | 34.09 | | 33.68 | 37.02 | | 36.52 | 36.52 | | 38.78 | **3** | |  |
| Rabbit 1 | Spleen | 33.85 | | 33.86 | 36.2 | | 37.78 | 38.96 | | 38.35 | **3** | |  |
| Rabbit 2 | Spleen | 33.52 | | 34.7 | 36.97 | | 38.84 | 37.68 | | 40 | **2** | |  |
| Rabbit 3 | Spleen | 37.1 | | 36.72 | 37.17 | | 39.05 | 37.89 | | N | **2** | |  |
| Hare 2 | Spleen | 34.62 | | 33.95 | 35.98 | | 36.48 | N | | 40 | **2** | |  |
| Hare 3 | Spleen | 36.41 | | 35.69 | 37.94 | | 37.64 | N | | 40 | **2** | |  |
| Hare 4 | Spleen | 33.58 | | 33.5 | 36.51 | | 36.68 | N | | 40 | **2** | |  |
| Rabbit 4 | Spleen | 37.08 | | 36.24 | 37.81 | | 38.45 | N | | N | **2** | |  |
| Hare 5 | Liver | 35.32 | | 35.99 | 37.33 | | 37.73 | N | | N | **2** | |  |
|  | Spleen | 37.82 | | 36.58 | 35.76 | | N | N | | N | **1** | |  |
| Rabbit 5 | Spleen | 37.81 | | 37.56 | 37.59 | | 38.13 | N | | N | **2** | |  |
| Hare 6 | Spleen | 38.19 | | 37.00 | 37.65 | | 37.68 | N | | N | **2** | |  |
| Hare 7 | Spleen | 35.1 | | 33.98 | 36.79 | | 38.04 | N | | N | **2** | |  |
| Rabbit 6 | Spleen | 37.03 | | 36.75 | N | | 37.75 | N | | N | **1** | |  |
| Hare 8 | Spleen | 35.62 | | 34.34 | N | | 37.12 | N | | N | **1** | |  |
| Rabbit 7 | Spleen | 36.23 | | 34.46 | N | | 37.79 | N | | N | **1** | |  |
| Rabbit 8 | Spleen | 36.72 | | 34.55 | N | | 35.98 | N | | N | **1** | |  |
| Rabbit 9 | Spleen | 37.31 | | 36.01 | N | | 37.97 | N | | N | **1** | |  |
| Hare 9 | Spleen | 36.11 | | 37.23 | N | | N | N | | N | **1** | |  |
| Hare 10 | Spleen | 36.45 | | 36.7 | N | | N | N | | N | **1** | |  |
| Rabbit 10 | Spleen | 36.47 | | 36.54 | N | | N | N | | N | **1** | |  |
| Hare 11 | Spleen | 35.85 | | 36.45 | N | | N | N | | N | **1** | |  |
| Hare 12 | Spleen | 35.1 | | 34.75 | N | | N | N | | N | **1** | |  |
| Rabbit 11 | Spleen | 36.6 | | 36.29 | N | | N | N | | N | **1** | |  |
| Hare 13 | Spleen | 37.07 | | 36.61 | N | | N | N | | N | **1** | |  |
| Hare 14 | Spleen | 37.07 | | 36.61 | N | | N | 40 | | 38.65 | **1** | |  |
|  | liver | 37.38 | | 36.77 | 37.85 | | N | N | | N | **1** | |  |
| Rabbit 12 | Liver | 37.14 | | 37.38 | N | | N | N | | N | **1** | |  |
|  | Spleen | N | | 40 | N | | N | N | | N |  | |  |
| Rabbit 13 | Liver | 37.23 | | 36.86 | N | | N | 40 | | N | **1** | |  |
|  | Spleen | N | | 40 | N | | N | N | | N |  | |  |
| Hare 15 | Spleen | 38.67 | | 37.38 | N | | N | N | | N |  | |  |
|  | Liver | 36.85 | | 35.93 | N | | N | N | | N | **1** | |  |
| Rabbit 14 | Spleen | 36.23 | | 36.14 | N | | N | N | | N | **1** | |  |
| Rabbit 15 | Spleen | 34.27 | | 34.02 | N | | N | N | | N | **1** | |  |
| Hare 16 | Liver | 36.47 | | 36.93 | N | | N | N | | N | **1** | |  |
|  | Spleen | 39.41 | | 40 | N | | N | N | | N |  | |  |

A qPCR test was considered positive only when two replicate tests were positive and a Ct value was <38 for ISFtu2-qPCR or <40 for Tul4-qPCR and Type B-qPCR.

* levels 1, 2, and 3 correspond respectively to probable, possible, and uncertain tularemia cases

N: no DNA amplification by the corresponding qPCR text

Hare 6, hare 12, and rabbit 13 were found dead
